# Comparative efficacy of exercise interventions for fall prevention in older adults with cognitive impairment: protocol for a systematic review and network meta-analysis

**DOI:** 10.3389/fmed.2026.1831444

**Published:** 2026-07-23

**Authors:** Jinsheng Lu, Shaojuan Huang, Yiyun Zeng, Danqin Li, Shaoying Song, Shuyan Chen, Jingli Chen, Qiaoping He, Jinyue Huang, Yao Hu

**Affiliations:** 1 Department of General Practice, The Eighth Clinical Medical College of Guangzhou University of Chinese Medicine, Foshan, Guangdong, China; 2 Department of General Practice, Foshan Hospital of Traditional Chinese Medicine, Foshan, Guangdong, China; 3 Department of Emergency, The Second Affiliated Hospital of Guangzhou University of Chinese Medicine, Guangzhou, Guangdong, China; 4 Department of Emergency, Guangdong Provincial Hospital of Chinese Medicine, Guangzhou, Guangdong, China; 5 Department of Emergency, The Second Clinical College of Guangzhou University of Chinese Medicine, Guangzhou, Guangdong, China

**Keywords:** accidental falls, cognitive impairment, dementia, exercise, mild cognitive impairment, network meta-analysis, older adults, systematic review

## Abstract

**Introduction:**

Older adults with mild cognitive impairment (MCI) or dementia have a substantially elevated risk of falls compared with cognitively intact peers, and the consequences of falls in this population are more severe. Multiple exercise modalities, including aerobic exercise, resistance training, balance training, mind-body exercise, multicomponent programs, dual-task training, and exergaming, have been investigated for fall prevention, yet the comparative efficacy of these interventions in cognitively impaired populations remains unclear. Existing evidence relies on pairwise meta-analyses that compare only two interventions at a time, limiting the ability to determine which exercise type is most effective. We will conduct a systematic review and network meta-analysis (NMA) to compare the efficacy of different exercise interventions for fall prevention in older adults with MCI or dementia.

**Methods:**

We will include randomized controlled trials (RCTs) comparing any structured exercise intervention (aerobic exercise, resistance training, balance training, mind-body exercise, multicomponent exercise, dual-task training, or exergaming) with each other or with control conditions in adults aged 60 years or older with MCI or dementia. The co-primary outcomes will be the number of fallers (odds ratio) and the rate of falls (incidence rate ratio). Secondary outcomes will include balance, mobility, cognitive function, fear of falling, and exercise-related adverse events. We will search PubMed, Embase, Cochrane CENTRAL, Web of Science, CINAHL and three Chinese-language databases without language restrictions. Risk of bias will be assessed using the Cochrane Risk of Bias 2 (RoB 2) tool. Data will be synthesized using a frequentist random-effects NMA, with a Bayesian NMA as a sensitivity analysis. Treatments will be ranked using P-scores, and the certainty of evidence will be assessed using the Confidence in Network Meta-Analysis (CINeMA) approach.

**Discussion:**

This NMA aims to provide a synthesis of the comparative efficacy of exercise interventions for fall prevention in cognitively impaired older adults. Should the available evidence prove sufficient, the resulting treatment rankings may help inform evidence-based exercise prescription in clinical practice.

**Systematic review registration:**

https://www.crd.york.ac.uk/PROSPERO/view/CRD420261303641, PROSPERO ID: CRD420261303641.

## 1 Introduction

Falls represent a leading cause of injury-related morbidity and mortality among older adults (1), with particularly devastating consequences for individuals with cognitive impairment. Older adults with mild cognitive impairment (MCI) or dementia experience fall rates approximately twice those of cognitively intact peers (2), and the sequelae of falls in this population — including hip fractures, traumatic brain injuries, prolonged hospitalization, and accelerated cognitive and functional decline — are substantially more severe (3). With the global prevalence of dementia projected to rise from an estimated 57 million cases in 2019 to over 150 million by 2050 (4), the burden of fall-related harm in cognitively impaired populations poses an escalating challenge to healthcare systems worldwide (5).

Exercise-based interventions have emerged as a cornerstone strategy for fall prevention in older adults (6). Various exercise modalities have been investigated, including aerobic exercise, resistance training, balance training, mind-body exercise (e.g., Tai Chi, yoga, Baduanjin), multicomponent exercise programs, dual-task training, and exergaming (7). Clinical guidelines recommend exercise as a first-line non-pharmacological intervention for fall prevention (8). However, the optimal exercise modality for cognitively impaired populations remains uncertain, as the mechanisms underlying fall risk in these individuals—such as impaired executive function, attentional deficits, and reduced dual-task capacity — may differ from those in cognitively intact older adults (9).

Several specific gaps in the current evidence base motivate the present review. First, existing systematic reviews on exercise and falls in cognitively impaired populations (10, 11) have relied exclusively on pairwise meta-analyses, which compare only two interventions at a time and cannot generate a unified ranking of all available exercise options. Second, the most comprehensive network meta-analyses of exercise for fall prevention, such as the Cochrane review by Sherrington et al. (6), have focused on the general older adult population without examining the cognitively impaired subgroup, and no existing NMA has simultaneously compared the full spectrum of exercise modalities — including emerging approaches such as dual-task training and exergaming — for fall prevention specifically in adults with MCI or dementia (9). Third, previous reviews in this population have predominantly examined balance or cognitive outcomes without prioritizing fall-specific endpoints as co-primary outcomes (12). Consequently, clinicians lack definitive guidance on which exercise modality to prescribe for fall prevention in this high-risk population.

To overcome these limitations, we will conduct a systematic review and NMA to simultaneously compare the efficacy of multiple exercise interventions for fall prevention in older adults with MCI or dementia. NMA extends traditional pairwise meta-analysis by integrating both direct and indirect evidence from RCTs, enabling the comparison of all available interventions and the generation of treatment rankings (13, 14). A fundamental assumption of NMA is transitivity, which requires that the distribution of potential effect modifiers is sufficiently similar across the different direct comparisons in the network (14). Because the included trials are expected to differ in cognitive status (MCI vs. dementia), the case definitions and screening thresholds used, residential settings, and the content and intensity of exercise protocols, this assumption may be challenged, and considerable clinical heterogeneity is anticipated. We therefore prespecify a structured transitivity assessment, subgroup analyses, and network meta-regression to evaluate and, where possible, account for these potential effect modifiers; these strategies are detailed in the Methods section. We further recognize that, given the anticipated sparse evidence base, statistical assessment of transitivity will have limited power; a non-significant result will therefore not be taken as confirmation that the assumption holds, and residual concerns will be reflected in the CINeMA certainty rating. The findings are expected to provide evidence-based guidance for the development of targeted exercise prescriptions for fall prevention in cognitively impaired older adults.

To assess the feasibility of the planned NMA, we conducted a preliminary scoping search in PubMed (search date: April 2026). Using a broad strategy combining terms for RCTs, accidental falls, exercise, and cognitive dysfunction, we identified 262 records. These records were screened by title and abstract, and potential duplicate reports of the same trial were identified manually by matching trial registration numbers, author names, study sites, and sample characteristics, and then linked to their primary report. After rapid title and abstract screening and accounting for multiple publications from the same trial, these records corresponded to an estimated 35–45 unique RCTs, the range reflects uncertainty in linking a small number of conference abstracts to their full reports. Head-to-head comparisons between different exercise modalities were identified in approximately five studies, suggesting that the network is likely to be predominantly star-shaped. Additional eligible studies are expected from the supplementary databases and gray literature sources included in our full search strategy. The implications of this anticipated network geometry are discussed in the Discussion section. Taken together, this preliminary search supports the feasibility of the planned synthesis, indicating that a sufficient number of eligible trials is likely to exist, while also confirming the sparse network geometry for which our prespecified analytical hierarchy is designed.

## 2 Methods

This protocol is reported according to the Preferred Reporting Items for Systematic Review and Meta-Analysis Protocols (PRISMA-P) statement (15) and will follow the PRISMA extension statement for network meta-analyses (PRISMA-NMA) (16). If amendments to the protocol are necessary, we will update the PROSPERO registration and include a clear report of any deviations in the published manuscript.

### 2.1 Eligibility criteria

#### 2.1.1 Study design

We will include RCTs with a parallel-group or crossover design. For crossover studies, only first-period data will be used to avoid carryover effects, which are particularly likely with exercise interventions due to the persistence of training adaptations (17); we anticipate that few crossover trials will be identified given the progressive nature of cognitive impairment in the target population. We recognize that restricting analysis to first-period data discards within-participant information and thereby reduces statistical efficiency; given the anticipated scarcity of crossover trials in this population, however, any resulting information loss is expected to be minimal. Cluster-randomized trials will be included with appropriate corrections applied to the effect estimates (14). Quasi-randomized, non-randomized, and observational studies will be excluded. No restrictions on sample size, follow-up duration, publication date, or language will be applied.

#### 2.1.2 Participants

We will include adults aged 60 years or older with cognitive impairment, defined as: (a) a clinical diagnosis of MCI or dementia (including Alzheimer's disease and vascular dementia), established using recognized diagnostic criteria such as the Petersen criteria (18), the National Institute on Aging–Alzheimer's Association criteria (19), or other validated standards; or (b) cognitive impairment documented by validated screening instruments using established cut-off scores [e.g., Mini-Mental State Examination (MMSE) score ≤ 26, Montreal Cognitive Assessment (MoCA) score ≤ 25, or equivalent thresholds on other validated tools]. We acknowledge that these screening thresholds are not universal: education-adjusted cut-off scores for instruments such as the MMSE and MoCA vary across countries and are influenced by educational attainment, language, and cultural context. Because such variation in case definition may act as a potential effect modifier and could threaten the transitivity assumption, the specific instrument and cut-off used in each study will be extracted and examined during the assessment of transitivity and in the sensitivity analyses described below [see analyses (b), (h), and (l)].

The inclusion of both MCI and dementia populations in the primary analysis reflects the current conceptualization of cognitive impairment as a clinical continuum. MCI and dementia share overlapping neuropathological substrates and fall risk mechanisms, including impaired executive function, attentional deficits, and reduced dual-task capacity (9). Moreover, many existing RCTs enroll mixed populations without reporting outcomes separately for each subgroup, which would preclude a fully stratified analysis in practice. We have therefore prespecified subgroup analyses stratified by cognitive impairment type (MCI vs. dementia) and a network meta-regression examining baseline cognitive severity as a study-level covariate to explore whether cognitive status modifies treatment effects (see Subgroup analyses and Sensitivity analyses). A sensitivity analysis restricted to studies using formal diagnostic criteria will further evaluate the impact of this broader inclusion approach (see Sensitivity analyses).

Participants may reside in community, residential care, or hospital settings. Studies specifically targeting populations with Parkinson's disease, acute stroke, or other neurological conditions that independently confer a high fall risk will be excluded. However, studies enrolling participants with MCI or dementia as the primary population will not be excluded solely because a minority of participants ( ≤ 20% of the study sample) have co-existing neurological conditions, provided these are not the primary inclusion criterion. This 20% threshold was specified a priori to balance the diagnostic purity of the target population against the inclusion of pragmatic, real-world trials that enroll a small minority of participants with incidental co-existing conditions; it operationalizes the convention that a sample can be regarded as predominantly representative of the target population when at least 80% of participants meet the core inclusion criterion. A more permissive threshold would risk admitting trials in which a substantial proportion of the treatment effect could be attributable to a distinct neurological population, thereby threatening transitivity, whereas a stricter threshold would unnecessarily exclude otherwise representative pragmatic trials and further reduce an already sparse evidence base. A sensitivity analysis excluding such studies will be conducted (see Sensitivity analyses). Studies involving mixed populations (e.g., cognitively impaired and cognitively intact participants) will be included only if outcomes for the cognitively impaired subgroup are reported separately.

#### 2.1.3 Interventions

Any structured exercise intervention administered as the primary experimental treatment will be eligible. Based on an a priori classification informed by established intervention taxonomies (20) and previous reviews (7), interventions will be categorized into the following nodes: (a) aerobic exercise (e.g., walking programs, cycling); (b) resistance training (e.g., elastic band exercises, machine-based strength training); (c) balance training (e.g., standing balance exercises, perturbation-based training); (d) mind-body exercise (e.g., Tai Chi, yoga, Baduanjin); (e) multicomponent exercise (i.e., programs combining two or more of the above categories); (f) dual-task training (i.e., exercise performed simultaneously with cognitive tasks); and (g) exergaming (i.e., interactive virtual reality or video game-based exercise). Single-session interventions will be excluded.

To ensure consistent classification, the following decision rules will be applied. First, each intervention will be classified according to its predominant exercise component, defined as the component occupying more than 50% of total session time; when session-time allocation is not reported, classification will be based on the primary stated aim of the intervention. Second, traditional mind-body modalities — including Tai Chi, yoga, qigong, Baduanjin, and Pilates — will be classified under the mind-body exercise node regardless of ancillary balance or strength components, consistent with the approach adopted by the Cochrane review on exercise for fall prevention (6). This classification reflects the shared core characteristics of these modalities, namely the integration of focused attention, controlled breathing, and slow movements within a meditative framework. Third, dual-task training will be assigned only when simultaneous cognitive-motor performance constitutes the primary training paradigm (i.e., occupying more than 50% of training sessions); otherwise, the intervention will be classified according to its dominant physical component. Fourth, classification disagreements between the two reviewers will be resolved by discussion and, if necessary, adjudication by a third reviewer; the final classification decision and its rationale will be documented for each included study.

To balance statistical power and clinical specificity, we will adopt a prespecified conditional node-definition strategy. For each candidate sub-node (e.g., Tai Chi separated from other mind-body exercises; multicomponent exercise split by dominant component), if the sub-node contains ≥3 independent studies and the network remains connected after splitting, the refined classification will be adopted in the primary analysis, with the broader classification retained as a sensitivity analysis; otherwise, the reverse arrangement will apply. The decision and its rationale will be documented transparently, and both classifications will be reported.

Where studies evaluate interventions combining exercise with non-exercise components (e.g., exercise plus pharmacological treatment), the study will be included only if the exercise effect can be isolated.

#### 2.1.4 Comparators

Eligible comparator conditions will include: (a) usual care; (b) health education or attention control (e.g., didactic sessions or social activities without structured exercise); (c) sham exercise (e.g., low-intensity stretching or gentle range-of-motion activities); and (d) no intervention (waiting list or no treatment). Head-to-head comparisons between two eligible exercise interventions will also be included. If the number of studies permits, these control conditions will be treated as separate nodes; otherwise, they will be collapsed into a single “non-exercise control” node, and a sensitivity analysis testing the impact of this decision will be performed.

#### 2.1.5 Outcomes

A fall will be defined as “an unexpected event in which the participant comes to rest on the ground, floor, or lower level,” consistent with the Prevention of Falls Network Europe (ProFaNE) consensus definition (21). Studies using alternative but clinically reasonable definitions will be included; the specific definition and ascertainment method used in each study will be recorded and explored as a potential source of heterogeneity.

The co-primary outcomes will be: (a) the number of fallers, defined as the proportion of participants experiencing one or more falls during the study period [dichotomous outcome; odds ratio (OR)]; and (b) the rate of falls, defined as the total number of fall events per unit of person-time [count outcome; incidence rate ratio (IRR)] (21). These two outcomes capture complementary dimensions of fall risk: the number of fallers reflects the breadth of risk reduction, whereas the rate of falls captures the impact on recurrent falling and overall fall burden.

Secondary outcomes will include: (a) balance, assessed using validated instruments such as the Berg Balance Scale (22) or the Tinetti Performance-Oriented Mobility Assessment (23); (b) mobility, assessed using the Timed Up and Go test (24); (c) global cognitive function, assessed using the MMSE (25) or the MoCA (26); (d) fear of falling, assessed using the Falls Efficacy Scale–International (27); and (e) exercise-related adverse events. Adverse events will be reported narratively and, where sufficient data are available, the proportion of participants experiencing any adverse event will be pooled. All outcomes will be assessed at the primary endpoint of each study, and data at all available time points will be extracted for the co-primary outcomes to explore the time course of treatment effects.

No formal multiplicity adjustment will be applied. We distinguish three potential sources of multiplicity and address each as follows. First, with respect to the two co-primary outcomes: this NMA is an exploratory evidence synthesis intended to generate treatment rankings rather than to test a single confirmatory hypothesis (28, 29), and the number of fallers and the rate of falls capture distinct but related dimensions of fall risk—the breadth of risk reduction and the burden of recurrent falling—rather than redundant tests of the same effect. They are therefore prespecified to be interpreted jointly, using the framework described below, and conclusions will not be drawn from either outcome in isolation. Second, with respect to the multiple pairwise comparisons inherent to the network, we will not apply per-comparison correction but will instead calibrate every conclusion to its CINeMA certainty rating and flag comparisons informed predominantly by indirect evidence as exploratory (see Data synthesis). Third, with respect to the numerous subgroup, meta-regression, and sensitivity analyses, the prespecified analytical hierarchy (see Hierarchy of analyses) designates only the primary and Bayesian-sensitivity NMAs as confirmatory; all other analyses are explicitly hypothesis-generating and cannot, by design, support comparative-superiority claims. We acknowledge that forgoing formal correction may modestly increase the risk of spurious findings; these safeguards are intended to mitigate that risk while preserving interpretive transparency.

### 2.2 Information sources and search strategy

We will search PubMed, Embase, Cochrane Central Register of Controlled Trials (CENTRAL), Web of Science, CINAHL and three Chinese-language databases. No restrictions on language, publication status, or document type will be applied. Specifically, preprints, conference abstracts, dissertations, and trial registry records with results will be eligible for inclusion, in accordance with Cochrane guidance to minimize publication bias (14). Non-peer-reviewed sources will be flagged during data extraction. To confirm: preprints fall under “publication status” and are eligible. In the primary analysis they will be pooled with peer-reviewed reports, while a pre-specified sensitivity analysis (Sensitivity analyses, item j) will separately exclude all non-peer-reviewed sources to assess their influence on the pooled estimates. Where the same trial is available as both a non-peer-reviewed and a peer-reviewed report, the peer-reviewed version will be used as the primary data source. The methodological quality of non-peer-reviewed reports will be appraised with RoB 2 in the same way as peer-reviewed studies, and any resulting impact on the certainty of evidence will be captured in the CINeMA “within-study bias” domain. We will also search clinical trial registries (http://ClinicalTrials.gov and the WHO International Clinical Trials Registry Platform) and ProQuest Dissertations & Theses Global to identify ongoing, completed but unpublished, or gray-literature studies. Reference lists of all included studies and relevant systematic reviews will be screened for additional eligible trials. The search strategy was constructed using four blocks of terms related to the population (e.g., cognitive impairment, dementia), interventions (e.g., exercise, physical activity, dual-task training, virtual reality), outcomes (e.g., accidental falls, postural balance), and study design (e.g., randomized controlled trial). These blocks were combined using Boolean operators AND/OR. The search terms included both MeSH Terms and free-text keywords (Title/Abstract) to ensure comprehensive retrieval. The detailed search strategies for all included databases are provided in Supplementary material 1. The search strategy will be peer-reviewed by a second information specialist using the Peer Review of Electronic Search Strategies (PRESS) checklist.

### 2.3 Study selection

Two reviewers will independently screen all identified titles and abstracts for potential eligibility. Full texts of potentially relevant records will be retrieved and independently assessed against the eligibility criteria by the same two reviewers. Inter-reviewer agreement at the full-text screening stage will be quantified using Cohen's kappa statistic. Disagreements will be resolved through discussion or by consulting a third senior reviewer. The study selection process will be documented in a PRISMA flow diagram (30), and a table of studies excluded at the full-text level with reasons will be provided.

### 2.4 Data extraction

Two reviewers will independently extract data using a pre-piloted standardized form. The following information will be collected: study characteristics (first author, year, country, setting, design, sample size), participant characteristics (age, sex, diagnosis, baseline cognitive status, disease severity), intervention details (type, frequency, intensity, duration, supervision), comparator details, and outcome data (means and standard deviations for continuous outcomes; event counts and denominators for dichotomous outcomes; event counts and person-time for rate outcomes).

To facilitate transparent evaluation of the intervention classification, we will additionally extract detailed intervention characteristics, including the specific exercise modality (e.g., Yang-style Tai Chi, Hatha yoga), session duration, frequency, total program duration, intensity prescription, progression strategy, supervision format, and delivery setting. These details will be presented in a supplementary Intervention Characteristics Table (Supplementary material 3).

In addition, we will extract indicators of intervention adherence and implementation fidelity, including: (a) the proportion of participants completing ≥75% of prescribed sessions; (b) mean attendance rate; (c) study drop-out rate; (d) supervision format (one-to-one, group, remote, or unsupervised); (e) provider qualification (e.g., physiotherapist, exercise physiologist, trained instructor, or caregiver); and (f) whether protocol-fidelity assessment (e.g., audio/video monitoring, fidelity checklists) was reported. These variables will be tabulated in Supplementary material 3 and used as candidate covariates in network meta-regression to explore their potential role as effect modifiers, when the number of studies permits.

For continuous outcomes, change-from-baseline values will be preferred over post-intervention endpoint values where both are available, as change scores may reduce between-study variability. When change-from-baseline data are not reported, endpoint values will be used; combining these formats within the same meta-analysis is acceptable under a random-effects model. Missing standard deviations will be imputed from standard errors, confidence intervals, or other available statistics using established methods (31). For all outcomes, data from intention-to-treat (ITT) analyses will be preferentially extracted over per-protocol results. When a study reports only per-protocol data, these will be used and the study will be flagged for sensitivity analysis. The number of participants randomized and the number analyzed in each trial arm will be recorded. For dichotomous outcomes, the primary analysis will assume that participants with missing outcome data experienced the event at the same rate as those with observed data (missing-at-random assumption). The impact of this assumption will be tested through worst-case and best-case sensitivity analyses (see Sensitivity analyses). Discrepancies will be resolved by discussion with a third reviewer. We will contact study authors when data are missing or unclear.

### 2.5 Risk of bias assessment

Two reviewers will independently assess the risk of bias of included studies using the Cochrane Risk of Bias 2 (RoB 2) tool (32) for each outcome. This tool evaluates five domains: bias arising from the randomization process, bias due to deviations from intended interventions, bias due to missing outcome data, bias in measurement of the outcome, and bias in selection of the reported result. Each domain will be judged as “low risk,” “some concerns,” or “high risk of bias,” and an overall judgment will be assigned. Disagreements will be resolved by discussion or consultation with a third reviewer.

### 2.6 Data synthesis

We will follow a two-step procedure. First, pairwise meta-analyses for all direct comparisons will be performed using a random-effects model with the restricted maximum likelihood (REML) estimator for the between-study heterogeneity variance (τ^2^) (33). Heterogeneity will be quantified using the I^2^ statistic and interpreted according to established thresholds: 0–40% might not be important, 30–60% may represent moderate heterogeneity, 50–90% may represent substantial heterogeneity, and 75–100% may represent considerable heterogeneity (34). The between-study variance (τ^2^) and 95% prediction intervals will also be reported.Within-node heterogeneity will additionally be reported separately for the mind-body exercise and multicomponent exercise nodes by computing node-specific τ^2^ for all direct comparisons contributing to these nodes, given the recognized diversity of interventions within these categories. We acknowledge that grouping diverse interventions into broad modality nodes inevitably introduces residual within-node heterogeneity. As described here and further in the Certainty of evidence section and the Discussion, this is addressed through node-specific reporting, pre-specified alternative-node-definition analyses, and CINeMA-based downgrading where appropriate. Second, if the assumptions of NMA are met, a frequentist random-effects NMA will be conducted using the REML estimator (14). A frequentist framework was chosen for the primary analysis because it provides transparent P-score-based treatment rankings, facilitates global inconsistency testing via the design-by-treatment interaction model, and ensures reproducibility through well-established analytical packages. A Bayesian sensitivity analysis will be performed to evaluate the robustness of the findings to the choice of analytical framework (see Sensitivity analyses).

The effect measure for the number of fallers will be the odds ratio (OR), which possesses desirable mathematical properties for NMA — including symmetry and consistent estimation regardless of arm — and ensures consistency of treatment effects across studies with different baseline event rates (14). We acknowledge that the OR may overestimate the relative risk when baseline event rates are high; therefore, results will additionally be presented as risk ratios (RRs) derived from the pooled OR estimates by back-transformation using the median control event rate, following the Cochrane Handbook approach (14). This is intended to aid clinical interpretation, as the risk ratio is more intuitively understood by clinicians than the odds ratio, while the odds ratio is retained as the primary modeling scale for its favorable mathematical properties in NMA. For the Bayesian sensitivity analysis, risk differences will also be reported. For the rate of falls, the incidence rate ratio (IRR) will be used. For continuous secondary outcomes, the standardized mean difference (SMD) will be used, as different measurement scales are expected. All effect estimates will be presented with their 95% confidence intervals (CIs).

The network geometry will be presented with a network plot, in which node sizes are proportional to the number of participants and edge widths are proportional to the number of studies. The seven exercise categories and the control conditions described above will form the network nodes. Network connectivity will be verified prior to conducting the NMA. If the network is disconnected, we will first attempt to restore connectivity by revising node definitions (e.g., collapsing similar categories); if the network remains disconnected, separate NMAs will be performed for each connected sub-network, supplemented by standard pairwise meta-analyses.

We will systematically evaluate network geometry prior to conducting the NMA. If the network is connected but predominantly star-shaped (i.e., most comparisons directed against a common comparator with few head-to-head trials), we will adopt the following approach: (a) pairwise meta-analysis results will be reported alongside NMA results; (b) a contribution matrix will identify the proportion of direct vs. indirect evidence for each comparison; (c) the certainty of evidence for comparisons relying predominantly on indirect evidence will be downgraded via the CINeMA “indirectness” domain; and (d) treatment rankings derived primarily from indirect evidence will be explicitly flagged as exploratory. The decision regarding the analytical approach will be made transparently and documented in the final manuscript.

The transitivity assumption will be assessed empirically through several complementary approaches (14, 35). First, a transitivity assessment table will be constructed listing prespecified potential effect modifiers for each direct comparison, including age, sex distribution, cognitive impairment type and severity, the screening instrument and diagnostic cut-off score used to define cognitive impairment (e.g., MMSE vs. MoCA), residential setting, intervention duration and frequency, supervision status, and fall ascertainment method (see Supplementary material 2). The distribution of these covariates will be examined through descriptive statistics and visual inspection to identify systematic imbalances. Second, when important imbalances are identified, their impact will be explored using network meta-regression (see Sensitivity analyses). Third, clinical and methodological similarity across studies within each comparison will be narratively assessed. If the transitivity assumption is judged to be substantially violated for specific comparisons, the certainty of evidence will be downgraded in the CINeMA “indirectness” domain.

Statistical agreement between direct and indirect evidence (i.e., consistency) will be evaluated locally using the node-splitting method (36) and globally using a design-by-treatment interaction test (37). In the presence of inconsistency, we will explore potential sources by examining the distribution of effect modifiers and will interpret findings cautiously.

If heterogeneity is not large and treatment effects are estimated with comparable uncertainty, treatments will be ranked using P-scores, the frequentist analog of the surface under the cumulative ranking curve (SUCRA) (38). P-scores range from 0 (worst) to 1 (best) and will be presented alongside league tables displaying all pairwise comparisons with 95% confidence intervals.

To prevent overinterpretation of rankings, the following rules will be applied: (a) P-scores reflect the mean extent of certainty that one treatment is better than another within the model and do not, by themselves, constitute evidence of statistically significant superiority; (b) when 95% confidence intervals of pairwise comparisons substantially overlap, differences in P-scores will be reported as uncertain rather than as evidence of differential efficacy; (c) when a comparison is informed predominantly (>70%) by indirect evidence, as identified by the contribution matrix, the corresponding ranking will be flagged as exploratory in all tables and figures; and (d) rankings will always be presented jointly with the certainty-of-evidence rating from CINeMA, and conclusions will be calibrated to the lowest certainty rating among the relevant comparisons.

When evaluating the comparative efficacy of interventions, the following prespecified framework will guide interpretation. (a) If an intervention is ranked favorably on both co-primary outcomes, this will be considered strong evidence supporting its efficacy. (b) If an intervention shows a statistically significant effect on one co-primary outcome but not the other, the interpretation will be restricted to the specific dimension of fall risk captured by the significant outcome. (c) In the event of discordant findings, the number of fallers will be prioritized for the primary interpretation. This prioritization is based on two considerations: the number of fallers is directly aligned with international fall prevention guidelines (8) and, as a dichotomous outcome, is less susceptible to distortion by recurrent fallers; furthermore, in cognitively impaired populations, accurate prospective counting of cumulative fall events is particularly challenging due to recall difficulties, whereas the ascertainment of whether at least one fall occurred is comparatively more reliable. The rate of falls will be discussed as a complementary indicator of overall fall burden. We further emphasize that, in dementia populations specifically, recurrent falls are a clinically critical phenomenon associated with progressive frailty, fear of falling, and institutionalization, and the rate of falls (IRR) provides a more sensitive measure of recurrent-fall burden than the number of fallers (OR). Therefore, when an intervention demonstrates a significant effect on the rate of falls but not on the number of fallers, this finding will not be interpreted as null but will be highlighted as evidence of a potential effect on fall recurrence, and discussed alongside the number-of-fallers result with equal interpretive weight in the context of recurrent-fall reduction. The prioritization of the number of fallers therefore applies primarily to headline summary conclusions and ranking outputs, not to the substantive clinical interpretation, which will integrate both outcomes throughout.

To operationalize this framework, results for both co-primary outcomes will be presented side by side. For each comparison, the finding will be classified as “concordant favorable” (significant benefit on both outcomes), “concordant null” (no significant effect on either), “partially discordant” (significant on one but not the other), or “fully discordant” (significant effects in opposite directions). The overall conclusion will be based primarily on the number of fallers, with the rate of falls providing supplementary context.

Data analysis will be conducted in R (version 4.4.0 or above; R Foundation for Statistical Computing, Vienna, Austria) using the packages meta (39) and netmeta (40).

### 2.7 Subgroup analyses

We will investigate potential sources of heterogeneity and inconsistency in the co-primary outcomes through prespecified subgroup analyses stratified by: (a) type of cognitive impairment (MCI vs. dementia), (b) residential setting (community vs. residential care vs. hospital), (c) intervention duration ( ≤ 12 weeks vs. >12 weeks), and (d) supervision status (supervised vs. unsupervised or home-based). For subgroup (a), when a study enrolls both MCI and dementia participants without reporting subgroup-specific data, classification will be based on the predominant population (i.e., >50% of enrolled participants); studies reporting subgroup-specific outcomes will contribute separate data to the relevant strata. These subgroup analyses will be conducted only when at least three studies per stratum are available. For the network meta-regression on cognitive impairment type and baseline cognitive severity (Sensitivity analyses, item h), we set a stricter threshold: at least 10 studies overall and at least 4 studies per covariate level are required for the meta-regression to be reported as a primary exploratory analysis. When these thresholds are not met, results will be reported descriptively only and explicitly labeled as hypothesis-generating, with no influence on the primary treatment rankings. A non-significant interaction in subgroup analyses or meta-regression should not be interpreted as evidence supporting transitivity, given the limited statistical power expected with sparse data; in such cases, the CINeMA “indirectness” domain will be downgraded if cognitive impairment type is unevenly distributed across direct comparisons. We further note that network meta-regression relies on study-level (aggregate) covariates and is therefore susceptible to aggregation (ecological) bias; combined with the small number of studies expected per covariate level, this means such analyses can reduce, but cannot eliminate, concerns about transitivity, and any residual imbalance—including in baseline mobility, executive function, supervision needs, and fall mechanisms—will be carried forward into the CINeMA “indirectness” rating rather than assumed to be resolved.

### 2.8 Sensitivity analyses

The robustness of the results for the co-primary outcomes will be assessed through the following prespecified sensitivity analyses: (a) excluding studies at high overall risk of bias; (b) excluding studies in which cognitive impairment was documented solely by screening instruments without a formal clinical diagnosis; (c) restricting to studies with a minimum follow-up of six months; (d) applying alternative node definitions, including separating Tai Chi from other mind-body exercises, reclassifying multicomponent programs according to their dominant exercise type, and testing different control node configurations (separate vs. collapsed) — as detailed in the conditional node-definition strategy (see Interventions), whichever classification (broader or more refined) is not selected for the primary analysis will be reported here as a sensitivity analysis; (e) restricting to studies reporting intention-to-treat results; (f) applying worst-case and best-case assumptions for missing participant outcome data in dichotomous outcomes; (g) refitting the primary NMA within a Bayesian random-effects framework using the gemtc package in R (interfacing with JAGS), with vague normal priors for treatment effect parameters and a half-normal prior for the between-study standard deviation — three Markov chains will be run with at least 100,000 iterations after a burn-in of 50,000 iterations, and convergence will be assessed using the Brooks-Gelman-Rubin diagnostic; (h) conducting a network meta-regression with cognitive impairment type (MCI vs. dementia) or baseline cognitive severity (mean MMSE or MoCA score) to explore whether cognitive impairment modifies relative treatment effects; (i) excluding studies in which co-existing neurological conditions affected more than 20% of the study sample; (j) restricting to studies using prospective fall ascertainment methods and separately excluding non-peer-reviewed sources (preprints, conference abstracts, and dissertations) to evaluate the impact of data quality and source type on the pooled estimates;(k) restricting to studies reporting an average adherence rate ≥75% (or, where adherence is not reported, restricting to studies with a drop-out rate < 20%), to assess whether implementation fidelity modifies the magnitude of treatment effects; (l) stratifying analyses by the screening instrument used to define cognitive impairment (e.g., MMSE vs. MoCA) and by the diagnostic cut-off score applied, and—where the number of studies permits—restricting to studies using education-adjusted thresholds, in order to assess whether the relative treatment effects are stable across differing case definitions.

### 2.9 Hierarchy of analyses and minimum evidence thresholds

Given that the anticipated evidence base (an estimated 35–45 RCTs across seven intervention nodes) may be sparse for several planned analyses, we prespecify the following analytical hierarchy to minimize overinterpretation of unstable estimates.

#### 2.9.1 Confirmatory analyses

Only the primary frequentist NMA on the co-primary outcomes and the Bayesian sensitivity NMA are designated as confirmatory; both require ≥2 studies per node and a connected network, otherwise we will revert to pairwise meta-analysis. Treatment rankings (*P*-scores) will be considered confirmatory only when direct evidence contributes ≥50% to the relevant comparisons; otherwise they will be reported but flagged as exploratory. The node definition used in the primary NMA will follow the conditional strategy described in the Interventions section; the alternative classification (broader or more refined, whichever was not selected for the primary analysis) will be reported as a sensitivity analysis.

#### 2.9.2 Exploratory analyses

All remaining analyses are explicitly hypothesis-generating, with the following thresholds: subgroup analyses require ≥3 studies per stratum; network meta-regression requires ≥10 studies overall and ≥4 per covariate level (otherwise reported descriptively); node-splitting requires ≥1 closed loop with ≥2 studies per edge (otherwise only the global inconsistency test is reported). Analyses not meeting their thresholds will not be performed.

#### 2.9.3 Handling of below-threshold results

Any results from analyses falling below their respective thresholds will be clearly labeled as exploratory in all tables, figures, and the abstract, and will not be used to support primary conclusions or revise treatment rankings. Only the two confirmatory analyses may inform conclusive statements on comparative efficacy; all others serve strictly as sources of hypotheses for future research.

### 2.10 Small-study effects and reporting bias

Small-study effects and potential publication bias will be examined for the co-primary outcomes using comparison-adjusted funnel plots (41). Contour-enhanced funnel plots will additionally be used in pairwise meta-analyses when at least ten studies are available for a given comparison.

### 2.11 Certainty of evidence

The certainty of evidence for the co-primary outcomes will be evaluated using the Confidence in Network Meta-Analysis (CINeMA) approach (42), which assesses six domains: within-study bias, reporting bias, indirectness, imprecision, heterogeneity, and incoherence. When within-node heterogeneity (assessed via node-specific τ^2^ and the diversity of intervention parameters in the Intervention Characteristics Table) is judged to be substantial, the corresponding comparisons will be downgraded in the CINeMA “heterogeneity” domain, and the resulting treatment rankings will be interpreted as modality-class rankings rather than rankings of any specific exercise sub-type. The overall certainty for each comparison will be categorized as very low, low, moderate, or high. These ratings will be used to qualify the interpretation of treatment rankings and to transparently communicate the reliability of the evidence.

## 3 Discussion

### 3.1 Strengths and contributions

Falls in older adults with cognitive impairment constitute a pressing clinical challenge, and exercise-based interventions offer a scalable, non-pharmacological approach to prevention. Our planned NMA is designed to synthesize evidence from all available RCTs on the comparative efficacy of multiple exercise modalities for fall prevention in older adults with MCI or dementia. By integrating both direct and indirect evidence, this analysis will enable simultaneous comparisons across all intervention types and generate clinically meaningful treatment rankings (13, 14).

### 3.2 Limitations

Several potential challenges and limitations should be acknowledged.

First, we anticipate considerable clinical heterogeneity across the included trials, arising from differences in the populations studied (MCI vs. various stages of dementia), residential settings, and exercise protocols. As detailed in the Participants section, our decision to include both MCI and dementia in the primary analysis is grounded in their shared neuropathological substrates and fall-risk mechanisms, and the limited feasibility of fully stratified analysis given that many primary trials enroll mixed populations without subgroup-specific reporting. We will address transitivity through prespecified subgroup analyses, network meta-regression on baseline cognitive severity, and a comprehensive transitivity assessment table comparing all prespecified effect modifiers across direct comparisons (Supplementary material 2). However, we acknowledge that statistical power for these analyses will be limited if studies per stratum are sparse, and a non-significant interaction will not be interpreted as evidence of transitivity. Where evidence of effect modification by cognitive status is uncertain, the CINeMA “indirectness” domain will be downgraded accordingly.

Second, our a priori classification of interventions into seven modality-based nodes, while pragmatically necessary to ensure sufficient statistical power and network connectivity, inevitably introduces residual within-node heterogeneity. For example, interventions classified under the “multicomponent exercise” node may vary substantially in the specific combination, proportion, and intensity of their component exercises, and “mind-body exercise” encompasses modalities as diverse as Tai Chi and yoga. More broadly, these mind-body sub-modalities — which also include qigong, Pilates, and Baduanjin — may differ appreciably in exercise intensity, balance demand, cognitive engagement, and supervision requirements, and the multicomponent node may likewise combine markedly different program structures. This within-node heterogeneity may attenuate between-node differences and reduce the precision of treatment rankings. To mitigate this limitation, we have pre-specified the following measures: (a) a conditional node-definition strategy (see Interventions) that adopts the more refined classification in the primary analysis when pre-specified thresholds are met, thereby maximizing the granularity of the node structure within the constraints of the available data; (b) reporting node-specific τ^2^ and 95% prediction intervals for all direct comparisons contributing to heterogeneous nodes; (c) downgrading the CINeMA “heterogeneity” domain when within-node τ^2^ is substantial; and (d) detailed tabulation of exercise parameters within each node in the Intervention Characteristics Table (Supplementary material 3) to facilitate transparent assessment of within-node variability. Accordingly, treatment rankings for nodes that cannot be further subdivided will be interpreted as modality-class rankings, and we will avoid claims of superiority for any single sub-modality unless directly supported by a refined-node analysis. We acknowledge, however, that even with this adaptive strategy, the anticipated sparse evidence base may not support fine-grained node splitting for all categories, and residual within-node heterogeneity may persist.

Third, the definition and ascertainment of falls varies across studies, with some relying on retrospective self-report and others using prospective fall calendars or electronic monitoring (43). Although we have adopted the ProFaNE consensus definition as the reference standard, we will document the fall ascertainment method for each included study and explore its impact in prespecified sensitivity analyses.

Fourth, blinding of participants and personnel is inherently difficult in exercise intervention trials, which may inflate the risk of performance and detection bias. We will assess the risk of bias for each outcome using the RoB 2 tool (32) and will conduct sensitivity analyses excluding studies at high risk of bias.

Fifth, adherence to exercise programs and implementation fidelity are recognized determinants of intervention efficacy and may be particularly relevant in cognitively impaired populations, where executive dysfunction, apathy, and caregiver burden can substantially reduce engagement. Inadequate or inconsistent reporting of adherence, supervision intensity, and instructor qualification in primary trials is a known limitation of the exercise-intervention literature. To address this, we have pre-specified the extraction of adherence and fidelity indicators (see Data extraction), their use as candidate covariates in network meta-regression, and a sensitivity analysis restricted to studies with adherence ≥75% or drop-out < 20% (Sensitivity analyses, item k). Where reporting is insufficient to support these analyses, we will flag this limitation explicitly when interpreting the rankings, and downgrade the CINeMA ‘within-study bias' or “heterogeneity” domains as appropriate.

Sixth, as reported in the Background, our preliminary scoping search identified an estimated 35–45 unique RCTs, with the majority comparing an active exercise intervention with usual care or no intervention and approximately five studies providing head-to-head comparisons between active modalities. On the basis of this preliminary evidence and previous systematic reviews (10, 12), we expect the network to be predominantly star-shaped, with most comparisons directed against usual care and few head-to-head comparisons between exercise interventions. In that scenario, many treatment comparisons will rely primarily on indirect evidence, and heterogeneity and incoherence may be difficult to evaluate comprehensively. To address this, we have pre-specified a transparent decision framework: pairwise meta-analysis results will be reported alongside NMA results, a contribution matrix will identify the evidence sources for each comparison, and treatment rankings derived primarily from indirect evidence will be explicitly flagged as exploratory. We will downgrade the certainty of evidence accordingly using CINeMA (42). If the network structure proves insufficient to support a valid NMA, pairwise meta-analyses will serve as the primary analytical output. We have also prespecified a strategy to address potential disconnectivity by revising node definitions and, if necessary, performing separate sub-network analyses (see Data synthesis).

### 3.3 Avoiding overinterpretation of unstable estimates

We acknowledge that our analytical plan is comprehensive relative to the anticipated evidence base. The hierarchy of analyses described in Methods operationalizes a prudent approach: only the primary NMA on the co-primary outcomes and the corresponding Bayesian sensitivity analysis are treated as confirmatory; subgroup analyses, meta-regressions, inconsistency assessments, and alternative node-definition sensitivity analyses are explicitly designated as hypothesis-generating. We will refrain from making causal or comparative-superiority claims based on these exploratory analyses, and the strength of all conclusions will be calibrated to the corresponding CINeMA certainty rating.

## 4 Conclusion

In conclusion, this protocol outlines a network meta-analysis comparing multiple exercise modalities for fall prevention in older adults with MCI or dementia. By integrating direct and indirect evidence within a single framework, the analysis is intended to assess, to the extent the evidence permits, the relative efficacy of these interventions in a population that existing NMAs have largely considered only as part of the general older adult population. Depending on the quantity and quality of the available evidence, the findings may help inform exercise prescription in clinical practice and identify priorities for future trials.
