# Supplementary material for: Comparative efficacy of exercise interventions for fall prevention in older adults with cognitive impairment: protocol for a systematic review and network meta-analysis
Source: Front Med (Lausanne). 2026 Jul 23;13:1831444. doi: 10.3389/fmed.2026.1831444 (PMC13441818; doi:10.3389/fmed.2026.1831444)
Supplement: Supplementary file 1 [file Supplementary_file_1.docx]

Additional file 1

Search Strategies for Each Database

The search strategies provided here represent the draft version developed by the review team. For the final version, input from an experienced health sciences librarian will be sought, and the strategies will undergo independent peer review following the PRESS guideline. All databases will be searched from their inception to the date the search is conducted.

**Database 1. PubMed (MEDLINE)**

**Block 1: Population**

1	Cognitive Dysfunction[MeSH Terms]

2	Dementia[MeSH Terms]

3	mild cognitive Impairment[Title/Abstract]

4	MCI[Title/Abstract]

5	senile dementia[Title/Abstract]

6	dementia[Title/Abstract]

7	neurocognitive disorder*[Title/Abstract]

8	cognitive declin*[Title/Abstract]

9	Alzheimer*[Title/Abstract]

10 memory loss[Title/Abstract]

11 memory impairment*[Title/Abstract]

12 (cognitive[Title/Abstract] AND (impair*[Title/Abstract] OR deficit*[Title/Abstract] OR dysfunction*[Title/Abstract]))

13 1 OR 2 OR 3 OR 4 OR 5 OR 6 OR 7 OR 8 OR 9 OR 10 OR 11 OR 12

**Block 2: Intervention**

14 Exercise Therapy[MeSH Terms]

15 Exercise[MeSH Terms]

16 Resistance Training[MeSH Terms]

17 Tai Ji[MeSH Terms]

18 Yoga[MeSH Terms]

19 strength training[Title/Abstract]

20 balance training[Title/Abstract]

21 walk*[Title/Abstract]

22 Pilates[Title/Abstract]

23 Baduanjin[Title/Abstract]

24 Qigong[Title/Abstract]

25 danc*[Title/Abstract]

26 multicomponent exercise[Title/Abstract]

27 physical training[Title/Abstract]

28 exercise[Title/Abstract]

29 physical activity[Title/Abstract]

30 resistance training[Title/Abstract]

31 aerobic*[Title/Abstract]

32 Tai Chi[Title/Abstract]

33 Tai Ji[Title/Abstract]

34 yoga[Title/Abstract]

35 gait training[Title/Abstract]

36 aquatic exercise[Title/Abstract]

37 hydrotherap*[Title/Abstract]

38 stretching[Title/Abstract]

39 rehabilitat*[Title/Abstract]

40 dual-task*[Title/Abstract]

41 dual task*[Title/Abstract]

42 cognitive-motor*[Title/Abstract]

43 motor-cognitive*[Title/Abstract]

44 Virtual Reality[MeSH Terms]

45 Video Games[MeSH Terms]

46 exergam*[Title/Abstract]

47 virtual reality[Title/Abstract]

48 video game*[Title/Abstract]

49 active video game*[Title/Abstract]

50 Kinect[Title/Abstract]

51 Wii[Title/Abstract]

52 14 OR 15 OR 16 OR 17 OR 18 OR 19 OR 20 OR 21 OR 22 OR 23 OR 24 OR 25 OR 26 OR 27 OR 28 OR 29 OR 30 OR 31 OR 32 OR 33 OR 34 OR 35 OR 36 OR 37 OR 38 OR 39 OR 40 OR 41 OR 42 OR 43 OR 44 OR 45 OR 46 OR 47 OR 48 OR 49 OR 50 OR 51

**Block 3: Outcome**

53 Accidental Falls[MeSH Terms]

54 Postural Balance[MeSH Terms]

55 accidental falls[Title/Abstract]

56 fall prevention[Title/Abstract]

57 postural balance[Title/Abstract]

58 postural control[Title/Abstract]

59 postural stability[Title/Abstract]

60 fall[Title/Abstract]

61 falls[Title/Abstract]

62 falling[Title/Abstract]

63 balance impairment*[Title/Abstract]

64 53 OR 54 OR 55 OR 56 OR 57 OR 58 OR 59 OR 60 OR 61 OR 62 OR 63

**Block 4: Study Design**

65 Randomized Controlled Trial[pt]

66 Controlled Clinical Trial[pt]

67 randomized[Title/Abstract]

68 randomised[Title/Abstract]

69 randomly[Title/Abstract]

70 trial[Title/Abstract]

71 RCT[Title/Abstract]

72 randomized controlled trial[Title/Abstract]

73 65 OR 66 OR 67 OR 68 OR 69 OR 70 OR 71 OR 72

74 animals[MeSH Terms] NOT humans[MeSH Terms]

75 73 NOT 74

**Combine**

76 13 AND 52 AND 64 AND 75

**Database 2. Embase (Ovid)**

**Block 1: Population**

1	exp cognitive defect/

2	exp dementia/

3	(mild cognitive adj2 impair*).ti,ab.

4	MCI.ti,ab.

5	senile dementia.ti,ab.

6	dementia.ti,ab.

7	neurocognitive disorder*.ti,ab.

8	cognitive declin*.ti,ab.

9	Alzheimer*.ti,ab.

10 memory loss.ti,ab.

11 memory impairment*.ti,ab.

12 (cognitive adj2 (impair* or deficit* or dysfunction*)).ti,ab.

13 1 or 2 or 3 or 4 or 5 or 6 or 7 or 8 or 9 or 10 or 11 or 12

**Block 2: Intervention**

14 exp kinesiotherapy/

15 exp exercise/

16 exp resistance training/

17 tai chi/

18 yoga/

19 strength training.ti,ab.

20 balance training.ti,ab.

21 walk*.ti,ab.

22 Pilates.ti,ab.

23 Baduanjin.ti,ab.

24 Qigong.ti,ab.

25 danc*.ti,ab.

26 multicomponent exercise.ti,ab.

27 physical training.ti,ab.

28 exercise.ti,ab.

29 physical activity.ti,ab.

30 resistance training.ti,ab.

31 aerobic*.ti,ab.

32 Tai Chi.ti,ab.

33 Tai Ji.ti,ab.

34 yoga.ti,ab.

35 gait training.ti,ab.

36 aquatic exercise.ti,ab.

37 hydrotherap*.ti,ab.

38 stretching.ti,ab.

39 rehabilitat*.ti,ab.

40 dual-task*.ti,ab.

41 dual task*.ti,ab.

42 cognitive-motor*.ti,ab.

43 motor-cognitive*.ti,ab.

44 virtual reality/

45 video game/

46 exergam*.ti,ab.

47 virtual reality.ti,ab.

48 video game*.ti,ab.

49 active video game*.ti,ab.

50 Kinect.ti,ab.

51 Wii.ti,ab.

52 14 or 15 or 16 or 17 or 18 or 19 or 20 or 21 or 22 or 23 or 24 or 25 or 26 or 27 or 28 or 29 or 30 or 31 or 32 or 33 or 34 or 35 or 36 or 37 or 38 or 39 or 40 or 41 or 42 or 43 or 44 or 45 or 46 or 47 or 48 or 49 or 50 or 51

**Block 3: Outcome**

53 falling/

54 body equilibrium/

55 accidental falls.ti,ab.

56 fall prevention.ti,ab.

57 postural balance.ti,ab.

58 postural control.ti,ab.

59 postural stability.ti,ab.

60 fall.ti,ab.

61 falls.ti,ab.

62 falling.ti,ab.

63 balance impairment*.ti,ab.

64 53 or 54 or 55 or 56 or 57 or 58 or 59 or 60 or 61 or 62 or 63

**Block 4: Study Design**

65 randomized controlled trial/

66 controlled clinical trial/

67 single blind procedure/

68 double blind procedure/

69 random*.ti,ab.

70 placebo.ti,ab.

71 (clinical adj2 trial).ti,ab.

72 trial.ti.

73 65 or 66 or 67 or 68 or 69 or 70 or 71 or 72

74 (exp animal/ or nonhuman/ or exp animal experiment/) not exp human/

75 73 not 74

**Combine**

76 13 and 52 and 64 and 75

**Database 3. Cochrane CENTRAL**

**Block 1: Population**

#1	MeSH descriptor: [Cognitive Dysfunction] explode all trees

#2	MeSH descriptor: [Dementia] explode all trees

#3	(mild cognitive NEAR/2 impair*):ti,ab,kw

#4	MCI:ti,ab,kw

#5	senile dementia:ti,ab,kw

#6	dementia:ti,ab,kw

#7	neurocognitive disorder*:ti,ab,kw

#8	cognitive declin*:ti,ab,kw

#9	Alzheimer*:ti,ab,kw

#10 memory loss:ti,ab,kw

#11 memory impairment*:ti,ab,kw

#12 (cognitive NEAR/2 (impair* OR deficit* OR dysfunction*)):ti,ab,kw

#13 #1 OR #2 OR #3 OR #4 OR #5 OR #6 OR #7 OR #8 OR #9 OR #10 OR #11 OR #12

**Block 2: Intervention**

#14 MeSH descriptor: [Exercise Therapy] explode all trees

#15 MeSH descriptor: [Exercise] explode all trees

#16 MeSH descriptor: [Resistance Training] explode all trees

#17 MeSH descriptor: [Tai Ji] explode all trees

#18 MeSH descriptor: [Yoga] explode all trees

#19 strength training:ti,ab,kw

#20 balance training:ti,ab,kw

#21 walk*:ti,ab,kw

#22 Pilates:ti,ab,kw

#23 Baduanjin:ti,ab,kw

#24 Qigong:ti,ab,kw

#25 danc*:ti,ab,kw

#26 multicomponent exercise:ti,ab,kw

#27 physical training:ti,ab,kw

#28 exercise:ti,ab,kw

#29 physical activity:ti,ab,kw

#30 resistance training:ti,ab,kw

#31 aerobic*:ti,ab,kw

#32 Tai Chi:ti,ab,kw

#33 Tai Ji:ti,ab,kw

#34 yoga:ti,ab,kw

#35 gait training:ti,ab,kw

#36 aquatic exercise:ti,ab,kw

#37 hydrotherap*:ti,ab,kw

#38 stretching:ti,ab,kw

#39 rehabilitat*:ti,ab,kw

#40 dual-task*:ti,ab,kw

#41 dual task*:ti,ab,kw

#42 cognitive-motor*:ti,ab,kw

#43 motor-cognitive*:ti,ab,kw

#44 MeSH descriptor: [Virtual Reality] explode all trees

#45 MeSH descriptor: [Video Games] explode all trees

#46 exergam*:ti,ab,kw

#47 virtual reality:ti,ab,kw

#48 video game*:ti,ab,kw

#49 active video game*:ti,ab,kw

#50 Kinect:ti,ab,kw

#51 Wii:ti,ab,kw

#52 #14 OR #15 OR #16 OR #17 OR #18 OR #19 OR #20 OR #21 OR #22 OR #23 OR #24 OR #25 OR #26 OR #27 OR #28 OR #29 OR #30 OR #31 OR #32 OR #33 OR #34 OR #35 OR #36 OR #37 OR #38 OR #39 OR #40 OR #41 OR #42 OR #43 OR #44 OR #45 OR #46 OR #47 OR #48 OR #49 OR #50 OR #51

**Block 3: Outcome**

#53 MeSH descriptor: [Accidental Falls] explode all trees

#54 MeSH descriptor: [Postural Balance] explode all trees

#55 accidental falls:ti,ab,kw

#56 fall prevention:ti,ab,kw

#57 postural balance:ti,ab,kw

#58 postural control:ti,ab,kw

#59 postural stability:ti,ab,kw

#60 fall:ti,ab,kw

#61 falls:ti,ab,kw

#62 falling:ti,ab,kw

#63 balance impairment*:ti,ab,kw

#64 #53 OR #54 OR #55 OR #56 OR #57 OR #58 OR #59 OR #60 OR #61 OR #62 OR #63

**Combine**

#65 #13 AND #52 AND #64

**Database 4. Web of Science Core Collection**

**Block 1: Population**

#1	TS=(mild cognitive NEAR/2 impair*)

#2	TS=MCI

#3	TS=(senile dementia)

#4	TS=dementia

#5	TS=(neurocognitive disorder*)

#6	TS=(cognitive declin*)

#7	TS=Alzheimer*

#8	TS=(memory loss)

#9	TS=(memory impairment*)

#10 TS=(cognitive NEAR/2 (impair* OR deficit* OR dysfunction*))

#11 #1 OR #2 OR #3 OR #4 OR #5 OR #6 OR #7 OR #8 OR #9 OR #10

**Block 2: Intervention**

#12 TS=(strength training)

#13 TS=(balance training)

#14 TS=walk*

#15 TS=Pilates

#16 TS=Baduanjin

#17 TS=Qigong

#18 TS=danc*

#19 TS=(multicomponent exercise)

#20 TS=(physical training)

#21 TS=exercise

#22 TS=(physical activity)

#23 TS=(resistance training)

#24 TS=aerobic*

#25 TS=(Tai Chi)

#26 TS=(Tai Ji)

#27 TS=yoga

#28 TS=(gait training)

#29 TS=(aquatic exercise)

#30 TS=hydrotherap*

#31 TS=stretching

#32 TS=rehabilitat*

#33 TS=dual-task*

#34 TS=(dual task*)

#35 TS=cognitive-motor*

#36 TS=motor-cognitive*

#37 TS=exergam*

#38 TS=(virtual reality)

#39 TS=(video game*)

#40 TS=(active video game*)

#41 TS=Kinect

#42 TS=Wii

#43 #12 OR #13 OR #14 OR #15 OR #16 OR #17 OR #18 OR #19 OR #20 OR #21 OR #22 OR #23 OR #24 OR #25 OR #26 OR #27 OR #28 OR #29 OR #30 OR #31 OR #32 OR #33 OR #34 OR #35 OR #36 OR #37 OR #38 OR #39 OR #40 OR #41 OR #42

**Block 3: Outcome**

#44 TS=(accidental falls)

#45 TS=(fall prevention)

#46 TS=(postural balance)

#47 TS=(postural control)

#48 TS=(postural stability)

#49 TS=fall

#50 TS=falls

#51 TS=falling

#52 TS=(balance impairment*)

#53 #44 OR #45 OR #46 OR #47 OR #48 OR #49 OR #50 OR #51 OR #52

**Block 4: Study Design**

#54 TS=(randomized OR randomised OR randomly)

#55 TS=("randomized controlled trial" OR "controlled clinical trial")

#56 TS=RCT

#57 TS=placebo

#58 TS=(clinical NEAR/2 trial)

#59 #54 OR #55 OR #56 OR #57 OR #58

**Combine**

#60 #11 AND #43 AND #53 AND #59

**Database 5. CINAHL**

**Block 1: Population**

S1	(MH "Cognition Disorders+")

S2	(MH "Dementia+")

S3	TI (mild cognitive N2 impair*) OR AB (mild cognitive N2 impair*)

S4	TI MCI OR AB MCI

S5	TI "senile dementia" OR AB "senile dementia"

S6	TI dementia OR AB dementia

S7	TI "neurocognitive disorder*" OR AB "neurocognitive disorder*"

S8	TI "cognitive declin*" OR AB "cognitive declin*"

S9	TI Alzheimer* OR AB Alzheimer*

S10 TI "memory loss" OR AB "memory loss"

S11 TI "memory impairment*" OR AB "memory impairment*"

S12 TI (cognitive N2 (impair* OR deficit* OR dysfunction*)) OR AB (cognitive N2 (impair* OR deficit* OR dysfunction*))

S13 S1 OR S2 OR S3 OR S4 OR S5 OR S6 OR S7 OR S8 OR S9 OR S10 OR S11 OR S12

**Block 2: Intervention**

S14 (MH "Therapeutic Exercise+")

S15 (MH "Exercise+")

S16 (MH "Resistance Training")

S17 (MH "Tai Chi")

S18 (MH "Yoga")

S19 (MH "Virtual Reality")

S20 (MH "Video Games")

S21 TI "strength training" OR AB "strength training"

S22 TI "balance training" OR AB "balance training"

S23 TI walk* OR AB walk*

S24 TI Pilates OR AB Pilates

S25 TI Baduanjin OR AB Baduanjin

S26 TI Qigong OR AB Qigong

S27 TI danc* OR AB danc*

S28 TI "multicomponent exercise" OR AB "multicomponent exercise"

S29 TI "physical training" OR AB "physical training"

S30 TI exercise OR AB exercise

S31 TI "physical activity" OR AB "physical activity"

S32 TI "resistance training" OR AB "resistance training"

S33 TI aerobic* OR AB aerobic*

S34 TI "Tai Chi" OR AB "Tai Chi"

S35 TI "Tai Ji" OR AB "Tai Ji"

S36 TI yoga OR AB yoga

S37 TI "gait training" OR AB "gait training"

S38 TI "aquatic exercise" OR AB "aquatic exercise"

S39 TI hydrotherap* OR AB hydrotherap*

S40 TI stretching OR AB stretching

S41 TI rehabilitat* OR AB rehabilitat*

S42 TI dual-task* OR AB dual-task*

S43 TI "dual task*" OR AB "dual task*"

S44 TI cognitive-motor* OR AB cognitive-motor*

S45 TI motor-cognitive* OR AB motor-cognitive*

S46 TI exergam* OR AB exergam*

S47 TI "virtual reality" OR AB "virtual reality"

S48 TI "video game*" OR AB "video game*"

S49 TI "active video game*" OR AB "active video game*"

S50 TI Kinect OR AB Kinect

S51 TI Wii OR AB Wii

S52 S14 OR S15 OR S16 OR S17 OR S18 OR S19 OR S20 OR S21 OR S22 OR S23 OR S24 OR S25 OR S26 OR S27 OR S28 OR S29 OR S30 OR S31 OR S32 OR S33 OR S34 OR S35 OR S36 OR S37 OR S38 OR S39 OR S40 OR S41 OR S42 OR S43 OR S44 OR S45 OR S46 OR S47 OR S48 OR S49 OR S50 OR S51

**Block 3: Outcome**

S53 (MH "Accidental Falls")

S54 (MH "Postural Balance")

S55 TI "accidental falls" OR AB "accidental falls"

S56 TI "fall prevention" OR AB "fall prevention"

S57 TI "postural balance" OR AB "postural balance"

S58 TI "postural control" OR AB "postural control"

S59 TI "postural stability" OR AB "postural stability"

S60 TI fall OR AB fall

S61 TI falls OR AB falls

S62 TI falling OR AB falling

S63 TI "balance impairment*" OR AB "balance impairment*"

S64 S53 OR S54 OR S55 OR S56 OR S57 OR S58 OR S59 OR S60 OR S61 OR S62 OR S63

**Block 4: Study Design**

S65 (MH "Clinical Trials+")

S66 PT "Clinical Trial"

S67 TI random* OR AB random*

S68 TI placebo OR AB placebo

S69 TI trial OR AB trial

S70 S65 OR S66 OR S67 OR S68 OR S69

**Combine**

S71 S13 AND S52 AND S64 AND S70

**CNKI**

(TKA='认知障碍' + '轻度认知障碍' + '认知功能障碍' + '认知损害' + '认知功能下降' + '认知功能减退' + '认知衰退' + '痴呆' + '老年痴呆' + '老年性痴呆' + '阿尔茨海默' + '血管性痴呆' + '路易体痴呆' + '额颞叶痴呆' + '神经认知障碍' + '记忆力减退' + '记忆障碍' + 'MCI') and (TKA='运动' + '运动疗法' + '运动训练' + '体育锻炼' + '体力活动' + '身体活动' + '力量训练' + '抗阻训练' + '弹力带训练' + '弹力带' + '平衡训练' + '步行' + '散步' + '太极拳' + '太极' + '瑜伽' + '八段锦' + '气功' + '五禽戏' + '舞蹈' + '有氧运动' + '有氧训练' + '多组分运动' + '综合运动' + '功能锻炼' + '功能性训练' + '步态训练' + '水中运动' + '水疗' + '拉伸' + '康复训练' + '双任务' + '双任务训练' + '认知运动训练' + '虚拟现实' + '体感游戏' + '电子游戏' + '普拉提' + '体操' + '健身操' + 'Wii' + 'Kinect') and (TKA='跌倒' + '摔倒' + '跌倒预防' + '预防跌倒' + '跌倒风险' + '防跌' + '姿势平衡' + '姿势控制' + '平衡能力' + '平衡功能' + '站立平衡' + '姿势稳定性' + '平衡障碍') and (TKA='随机对照试验' + '随机对照' + '随机' + '随机分配' + '临床试验' + 'RCT')

**WangFang Data**

主题:("认知障碍" OR "轻度认知障碍" OR "认知功能障碍" OR "认知损害" OR "认知功能下降" OR "认知功能减退" OR "认知衰退" OR "痴呆" OR "老年痴呆" OR "老年性痴呆" OR "阿尔茨海默" OR "血管性痴呆" OR "路易体痴呆" OR "额颞叶痴呆" OR "神经认知障碍" OR "记忆力减退" OR "记忆障碍" OR "MCI") and 主题:("运动" OR "运动疗法" OR "运动训练" OR "体育锻炼" OR "体力活动" OR "身体活动" OR "力量训练" OR "抗阻训练" OR "弹力带训练" OR "弹力带" OR "平衡训练" OR "步行" OR "散步" OR "太极拳" OR "太极" OR "瑜伽" OR "八段锦" OR "气功" OR "五禽戏" OR "舞蹈" OR "有氧运动" OR "有氧训练" OR "多组分运动" OR "综合运动" OR "功能锻炼" OR "功能性训练" OR "步态训练" OR "水中运动" OR "水疗" OR "拉伸" OR "康复训练" OR "双任务" OR "双任务训练" OR "认知运动训练" OR "虚拟现实" OR "体感游戏" OR "电子游戏" OR "普拉提" OR "体操" OR "健身操" OR "Wii" OR "Kinect") and 主题:("跌倒" OR "摔倒" OR "跌倒预防" OR "预防跌倒" OR "跌倒风险" OR "防跌" OR "姿势平衡" OR "姿势控制" OR "平衡能力" OR "平衡功能" OR "站立平衡" OR "姿势稳定性" OR "平衡障碍") and 主题:("随机对照试验" OR "随机对照" OR "随机" OR "随机分配" OR "临床试验" OR "RCT")

**VIP**

(M=("认知障碍" OR "轻度认知障碍" OR "认知功能障碍" OR "认知损害" OR "认知功能下降" OR "认知功能减退" OR "认知衰退" OR "痴呆" OR "老年痴呆" OR "老年性痴呆" OR "阿尔茨海默" OR "血管性痴呆" OR "路易体痴呆" OR "额颞叶痴呆" OR "神经认知障碍" OR "记忆力减退" OR "记忆障碍" OR "MCI") OR R=("认知障碍" OR "轻度认知障碍" OR "认知功能障碍" OR "认知损害" OR "认知功能下降" OR "认知功能减退" OR "认知衰退" OR "痴呆" OR "老年痴呆" OR "老年性痴呆" OR "阿尔茨海默" OR "血管性痴呆" OR "路易体痴呆" OR "额颞叶痴呆" OR "神经认知障碍" OR "记忆力减退" OR "记忆障碍" OR "MCI")) AND (M=("运动" OR "运动疗法" OR "运动训练" OR "体育锻炼" OR "体力活动" OR "身体活动" OR "力量训练" OR "抗阻训练" OR "弹力带训练" OR "弹力带" OR "平衡训练" OR "步行" OR "散步" OR "太极拳" OR "太极" OR "瑜伽" OR "八段锦" OR "气功" OR "五禽戏" OR "舞蹈" OR "有氧运动" OR "有氧训练" OR "多组分运动" OR "综合运动" OR "功能锻炼" OR "功能性训练" OR "步态训练" OR "水中运动" OR "水疗" OR "拉伸" OR "康复训练" OR "双任务" OR "双任务训练" OR "认知运动训练" OR "虚拟现实" OR "体感游戏" OR "电子游戏" OR "普拉提" OR "体操" OR "健身操" OR "Wii" OR "Kinect") OR R=("运动" OR "运动疗法" OR "运动训练" OR "体育锻炼" OR "体力活动" OR "身体活动" OR "力量训练" OR "抗阻训练" OR "弹力带训练" OR "弹力带" OR "平衡训练" OR "步行" OR "散步" OR "太极拳" OR "太极" OR "瑜伽" OR "八段锦" OR "气功" OR "五禽戏" OR "舞蹈" OR "有氧运动" OR "有氧训练" OR "多组分运动" OR "综合运动" OR "功能锻炼" OR "功能性训练" OR "步态训练" OR "水中运动" OR "水疗" OR "拉伸" OR "康复训练" OR "双任务" OR "双任务训练" OR "认知运动训练" OR "虚拟现实" OR "体感游戏" OR "电子游戏" OR "普拉提" OR "体操" OR "健身操" OR "Wii" OR "Kinect")) AND (M=("跌倒" OR "摔倒" OR "跌倒预防" OR "预防跌倒" OR "跌倒风险" OR "防跌" OR "姿势平衡" OR "姿势控制" OR "平衡能力" OR "平衡功能" OR "站立平衡" OR "姿势稳定性" OR "平衡障碍") OR R=("跌倒" OR "摔倒" OR "跌倒预防" OR "预防跌倒" OR "跌倒风险" OR "防跌" OR "姿势平衡" OR "姿势控制" OR "平衡能力" OR "平衡功能" OR "站立平衡" OR "姿势稳定性" OR "平衡障碍")) AND (M=("随机对照试验" OR "随机对照" OR "随机" OR "随机分配" OR "临床试验" OR "RCT") OR R=("随机对照试验" OR "随机对照" OR "随机" OR "随机分配" OR "临床试验" OR "RCT"))
