# Supplementary material for: Comparative efficacy of exercise interventions for fall prevention in older adults with cognitive impairment: protocol for a systematic review and network meta-analysis
Source: Front Med (Lausanne). 2026 Jul 23;13:1831444. doi: 10.3389/fmed.2026.1831444 (PMC13441818; doi:10.3389/fmed.2026.1831444)
Supplement: Supplementary file 2 [file Supplementary_file_2.docx]

Additional file 2: Transitivity Assessment Table Template

This table will be completed in the full review to evaluate the transitivity assumption. For each direct comparison available in the network, the distribution of prespecified potential effect modifiers will be summarized. Values presented will be the median (range) or weighted mean across studies within each comparison. Systematic imbalances across comparisons may threaten the validity of indirect estimates and will be explored through network meta-regression or addressed by downgrading the certainty of evidence in the CINeMA 'indirectness' domain.

The first row of each table (shown in italics) presents hypothetical, illustrative data to demonstrate the intended format; it does not represent findings from any actual study. All cells are intentionally left blank in this protocol template and will be populated during data extraction in the completed review.

Table A. Participant Characteristics by Direct Comparison

| Direct Comparison | No. of Studies (k) | Total (N) | Mean Age, years (mean, range) | % Female (mean, range) | Cognitive Impairment Type (MCI / Dementia / Mixed) | Mean MMSE or MoCA score (median, range) | Severity Stage (Mild / Moderate / Severe) | Setting (Community / Residential / Hospital) |
| --- | --- | --- | --- | --- | --- | --- | --- | --- |
| *Illustrative example (hypothetical data)* | *6* | *412* | *74.3 (68–82)* | *63 (55–71)* | *MCI* | *MMSE 26 (24–28)* | *Mild* | *Community* |
| Active intervention vs Non-exercise control | | | | | | | | |
| Aerobic exercise vs Non-exercise control |  |  |  |  |  |  |  |  |
| Resistance training vs Non-exercise control |  |  |  |  |  |  |  |  |
| Balance training vs Non-exercise control |  |  |  |  |  |  |  |  |
| Mind-body exercise vs Non-exercise control |  |  |  |  |  |  |  |  |
| Multicomponent exercise vs Non-exercise control |  |  |  |  |  |  |  |  |
| Dual-task training vs Non-exercise control |  |  |  |  |  |  |  |  |
| Exergaming vs Non-exercise control |  |  |  |  |  |  |  |  |
| *Head-to-head comparisons between active interventions****^a^*** | | | | | | | | |
| Mind-body exercise vs Balance training |  |  |  |  |  |  |  |  |
| Multicomponent exercise vs Balance training |  |  |  |  |  |  |  |  |
| Dual-task training vs Multicomponent exercise |  |  |  |  |  |  |  |  |
| Exergaming vs Balance training |  |  |  |  |  |  |  |  |
| [Additional rows to be added as identified] |  |  |  |  |  |  |  |  |

Abbreviations: MCI = mild cognitive impairment; MMSE = Mini-Mental State Examination; MoCA = Montreal Cognitive Assessment.

Data source: All values will be extracted from the studies included in the completed systematic review. Cells are intentionally left blank in this protocol template and will be populated during data extraction. Entries will be reported as median (range) for continuous variables and n (%) for categorical variables, unless otherwise specified.

^a^Head-to-head comparisons listed here are illustrative; the final set of rows will be determined by the studies identified during the systematic search. Only comparisons with at least one contributing study will be included in the completed table.

Table B. Intervention and Methodological Characteristics by Direct Comparison

| Direct Comparison | Intervention Duration, weeks (median, range) | Frequency, sessions/week (median, range) | Session Duration, minutes (median, range) | Total Dose, hours (median, range) | Follow-up Duration, months (median, range) | Exercise Intensity (Low / Moderate / High / Not reported), n (%) | Supervision (Supervised / Unsupervised / Mixed), n (%) | Fall Definition Provided (Yes / No), n (%) | Fall Ascertainment Method (Prospective diary / Retrospective recall / Medical records / Other), n (%) | Screening Instrument / Diagnostic Cut-off |
| --- | --- | --- | --- | --- | --- | --- | --- | --- | --- | --- |
| Illustrative example (hypothetical data) | 12 (8–24) | 3 (2–5) | 45 (30–60) | 27 (16–48) | 6 (3–12) | Moderate, 4 (67) | Supervised, 5 (83) | Yes, 6 (100) | Prospective diary, 4 (67) | MMSE < 26; MoCA < 26 |
| Active intervention vs Non-exercise control | | | | | | | | | |  |
| Aerobic exercise vs Non-exercise control |  |  |  |  |  |  |  |  |  |  |
| Resistance training vs Non-exercise control |  |  |  |  |  |  |  |  |  |  |
| Balance training vs Non-exercise control |  |  |  |  |  |  |  |  |  |  |
| Mind-body exercise vs Non-exercise control |  |  |  |  |  |  |  |  |  |  |
| Multicomponent exercise vs Non-exercise control |  |  |  |  |  |  |  |  |  |  |
| Dual-task training vs Non-exercise control |  |  |  |  |  |  |  |  |  |  |
| Exergaming vs Non-exercise control |  |  |  |  |  |  |  |  |  |  |
| *Head-to-head comparisons between active interventions****^a^*** | | | | | | | | | |  |
| Mind-body exercise vs Balance training |  |  |  |  |  |  |  |  |  |  |
| Multicomponent exercise vs Balance training |  |  |  |  |  |  |  |  |  |  |
| Dual-task training vs Multicomponent exercise |  |  |  |  |  |  |  |  |  |  |
| Exergaming vs Balance training |  |  |  |  |  |  |  |  |  |  |
| [Additional rows to be added as identified] |  |  |  |  |  |  |  |  |  |  |

Abbreviations: RPE = Rating of Perceived Exertion; wk = week; wks = weeks; min = minutes; CERT = Consensus on Exercise Reporting Template; — = not applicable.

Data source: All values will be extracted from the studies included in the completed systematic review. Cells are intentionally left blank in this protocol template and will be populated during data extraction. Entries will be reported as median (range) for continuous variables and n (%) for categorical variables, unless otherwise specified.

^a^Professional background of the person delivering the intervention (e.g., physiotherapist, exercise physiologist, occupational therapist, certified instructor, nurse, research assistant, or not reported), corresponding to CERT item 1.

RPE = Rating of Perceived Exertion; wk = week; wks = weeks; min = minutes; — = not applicable.
