# Supplementary material for: Comparative efficacy of exercise interventions for fall prevention in older adults with cognitive impairment: protocol for a systematic review and network meta-analysis
Source: Front Med (Lausanne). 2026 Jul 23;13:1831444. doi: 10.3389/fmed.2026.1831444 (PMC13441818; doi:10.3389/fmed.2026.1831444)
Supplement: Supplementary file 3 [file Supplementary_file_3.docx]

Additional file 3: Intervention Characteristics Table Template

This table will be completed in the full review to provide a transparent record of the intervention characteristics for each included study and to justify the network meta-analysis (NMA) node classification. Each row represents one study arm, including both intervention and control arms. Studies with multiple arms will have multiple rows. Readers may use this table to evaluate the appropriateness of the node assignments, to assess clinical heterogeneity within each node, and to verify the basis for any node re-classification conducted in sensitivity analyses (e.g., splitting the mind-body exercise node into Tai Chi, Yoga, and other mind-body modalities).

Intervention descriptions follow the Template for Intervention Description and Replication (TIDieR) checklist and the Consensus on Exercise Reporting Template (CERT) where applicable. The seven a priori intervention nodes are: (1) aerobic exercise, (2) resistance training, (3) balance training, (4) mind-body exercise, (5) multicomponent exercise, (6) dual-task training, and (7) exergaming. Control conditions are classified as: usual care, health education / attention control, sham exercise, or waitlist / no intervention.

**Table A. Intervention Description and NMA Node Assignment**

| **Study ID** | **Arm** | **NMA Node** | **Specific Modality** | **Brief Intervention Description^a^** | **Co-interventions^b^** | **Node Classification Rationale** |
| --- | --- | --- | --- | --- | --- | --- |
| *e.g., Li 2019* | *Intervention 1* | *Mind-body exercise* | *Tai Chi* | *Yang-style 24-form Tai Chi; progressive introduction of forms over 12 weeks* | *None* | *Primary component is Tai Chi, classified under mind-body exercise per protocol definition (lines 177–179)* |
| *e.g., Li 2019* | *Control* | *Non-exercise control (usual care)* | *—* | *Standard medical care; participants asked to maintain habitual activity level* | *Continued cholinesterase inhibitor therapy* | *No structured exercise component; classified as usual care* |
| *e.g., Smith 2021* | *Intervention 1* | *Multicomponent exercise* | *Combined balance + resistance* | *Sessions included 20 min balance training and 20 min lower-limb resistance exercises* | *Weekly cognitive stimulation group sessions* | *Contains ≥2 distinct exercise types with no single dominant component (≥60% of session); classified as multicomponent per protocol definition (lines 173–176)* |
| *e.g., Smith 2021* | *Intervention 2* | *Balance training* | *Otago Exercise Programme* | *Otago home-based programme: balance and strength exercises prescribed by physiotherapist* | *Weekly cognitive stimulation group sessions* | *≥60% of session time devoted to balance exercises; classified as balance training* |
| ...... |  |  |  |  |  |  |

^a^ One-to-two sentence summary of the intervention content, following the TIDieR "what" item.
^b^ Any additional treatments (pharmacological or non-pharmacological) delivered alongside the exercise intervention to both or either arm.

**Table B. Intervention Dosage and Delivery Details**

| **Study ID** | **Arm** | **Session Duration (min)** | **Frequency (sessions/wk)** | **Total Duration (wks)** | **Total Dose (hours)** | **Intensity Prescription** | **Progression Strategy** | **Delivered by^a^** | **Supervision Format (Individual / Group / Remote / Mixed)** | **Delivery Setting (Community / Residential / Hospital / Home)** | **Adherence Rate (%)** |
| --- | --- | --- | --- | --- | --- | --- | --- | --- | --- | --- | --- |
| *e.g., Li 2019* | *Intervention 1* | *60* | *3* | *24* | *72* | *Low-to-moderate (self-paced)* | *New forms added every 2 wks* | *Certified Tai Chi instructor* | *Group* | *Community centre* | *82* |
| *e.g., Li 2019* | *Control* | *—* | *—* | *24* | *—* | *—* | *—* | *—* | *—* | *—* | *—* |
| *e.g., Smith 2021* | *Intervention 1* | *40* | *2* | *16* | *21.3* | *Moderate (RPE 12–14)* | *Resistance load increased when 2 × 10 reps completed* | *Physiotherapist* | *Group* | *Residential care facility* | *75* |
| *e.g., Smith 2021* | *Intervention 2* | *30* | *3* | *16* | *24* | *Not reported* | *Difficulty increased at physiotherapist visits* | *Physiotherapist (initial); self-directed* | *Mixed* | *Home* | *68* |
| ...... |  |  |  |  |  |  |  |  |  |  |  |

^a^ Professional background of the person delivering the intervention (e.g., physiotherapist, exercise physiologist, occupational therapist, certified instructor, nurse, research assistant, or not reported), corresponding to CERT item 1.
RPE = Rating of Perceived Exertion; wk = week; wks = weeks; min = minutes; — = not applicable.
